# Social epidemiology of cardiometabolic risk factors in early adolescents

**DOI:** 10.1016/j.ijcrp.2025.200382

**Published:** 2025-03-06

**Authors:** Jason M. Nagata, Christiane K. Helmer, Jennifer H. Wong, Seohyeong Lee, Sydnie K. Domingue, Patrick Low, Abubakr A.A. Al-shoaibi, Joan E. Shim, Kyle T. Ganson, Alexander Testa, Orsolya Kiss, Holly C. Gooding, Erin E. Dooley, Kelley Pettee Gabriel, Fiona C. Baker

**Affiliations:** a Department of Pediatrics, University of California, 550 16th Street, 4th Floor, Box 0503, San Francisco, CA, 94143, USA; b Factor-Inwentash Faculty of Social Work, University of Toronto, 246 Bloor St W, Toronto, ON, M5S 1V4, Canada; c Department of Management, Policy and Community Health, University of Texas Health Science Center at Houston, 1200 Pressler Street, Houston, TX, 77030, USA; d Center for Health Sciences, SRI International, 333 Ravenswood Ave, Menlo Park, CA, 94025, USA; e Division of General Pediatrics and Adolescent Medicine, Department of Pediatrics, Emory University School of Medicine and Children’s Healthcare of Atlanta, 2015 Uppergate Drive, Atlanta, GA, 30322, USA; f Department of Epidemiology, University of Alabama at Birmingham, 1665 University Boulevard, Birmingham, AL, 35233, USA; g School of Physiology, University of the Witwatersrand, 1 Jan Smuts Ave, Braamfontein, Johannesburg, 2000, South Africa

**Keywords:** Cardiovascular disease, Cholesterol, Diabetes, Adolescent

## Abstract

**Background:**

To estimate associations between sociodemographic factors and cardiometabolic risk factors among a demographically diverse sample of U.S. adolescents aged 10–14 years.

**Methods:**

This study analyzed data from the Adolescent Brain Cognitive Development (ABCD) Study (N = 1412), Years 2 and 3 (2018–2021). Cardiometabolic risk factors including hemoglobin A1c and cholesterol (total cholesterol (TC), high-density lipoprotein cholesterol (HDL-C), and non-HDL-C) were assessed. Multivariable linear regression models were conducted to estimate the associations between sociodemographic factors (age, sex, race and ethnicity, household income, and parental education) and cardiometabolic risk factors (hemoglobin A1c, TC, HDL-C, and non-HDL-C).

**Results:**

The average hemoglobin A1c level was 5.2 % (±0.4 %), the average TC level was 156.6 (±28.9) mg/dL, and the average HDL-C level was 56.0 (±12.9) mg/dL. Out of our sample, 0.5 % had diabetes (hemoglobin A1c ≥ 6.5 %), 7.6 % had high TC (≥200 mg/dL), and 7.4 % had low HDL-C (<40 mg/dL). Older age was associated with lower TC, HDL-C, and non-HDL-C levels. Male sex was associated with higher hemoglobin A1c (beta coefficient [B] 0.04; 95 % confidence interval [CI], 0.00, 0.08; p = 0.037) and lower TC (B −3.14; 95 % CI, −6.17, −0.11; p = 0.042) compared to female sex. Black and Native American race and ethnicity were associated with higher hemoglobin A1c compared to White race. Higher household income was associated with higher TC and HDL-C.

**Conclusion:**

This study of a diverse population of early adolescents identified sociodemographic differences in hemoglobin A1c and cholesterol levels that can inform clinical and public health interventions.

## Highlights

• Older age was associated with lower total, HDL, and non-HDL cholesterol.
• Male sex was associated with higher hemoglobin A1c and lower total cholesterol.
• Black and Native American race/ethnicity were associated with higher hemoglobin A1c.
• Higher household income was associated with total cholesterol and HDL cholesterol.

## 1 Introduction

The prevalence of childhood cardiometabolic risk and diseases such as dyslipidemia (low high-density lipoprotein cholesterol (HDL-C), high low-density lipoprotein cholesterol (LDL-C), and high triglycerides), and type 2 diabetes has increased in recent years [1,2]. In the United States (U.S.), 7.4 % of children aged 6–19 years during 2011–2014 had high total cholesterol (TC), composed of LDL-C, HDL-C, and triglycerides, and 0.067 % aged 10–19 in 2017 had type 2 diabetes [1,3]. Earlier age at onset and greater number of cumulative years with diabetes and dyslipidemia predispose adolescents to cardiovascular morbidity in adulthood through atherosclerotic vascular changes [4]. Therefore, identification and management of risk factors for cardiometabolic disease during adolescence is an important ongoing area of investigation.

Social epidemiology explores how various demographic and socioeconomic factors impact health outcomes to gain insight into health disparities [5]. Mechanistically, sociodemographic factors may result in cardiometabolic risk through influences on diet, physical activity, access to healthcare, and racial minority stress [[6], [7], [8]]. Prior research has identified elevated hemoglobin A1c levels, a biomarker used in the diagnosis and monitoring of diabetes, among racial minority adolescents with overweight/obesity, including Black adolescents, in contrast to their non-Hispanic White counterparts [9]. Additionally, female adolescents have been found to have higher levels of TC, LDL-C, and non-HDL-C compared to male adolescents likely in the setting of chronic physiologic and pubertal processes [10]. Low household income is associated with a higher risk of developing type 2 diabetes in adolescence [11] and higher blood pressure in early adolescence [12]. Although prior research has examined sociodemographic factors of metabolic disease in adolescents [7,[13], [14], [15]], there is a paucity in the literature describing sociodemographic associations with cholesterol and diabetes risk specifically in early adolescence, which is an important life stage for the development of cardiometabolic risk [16]. To address this gap, we examined the association between sociodemographic factors and cardiometabolic disease risk factors in a large, national, diverse sample of early U.S. adolescents aged 10–14 years.

## 2 Materials and methods

We examined data from Year 2 (2018–2020) and Year 3 (2019–2021) of the Adolescent Brain and Cognitive Development (ABCD) Study, the largest longitudinal study of brain health and development in the U.S. Across 21 demographically diverse sites from four U.S. regions, 11,875 children were recruited in 2016–2018 (baseline; Year 0). The baseline sample of participants was recruited through elementary schools, which were selected via a stratified, multi-stage probability sampling within the catchment areas. Gender, race and ethnicity, socioeconomic status, and urbanicity were used to select schools to minimize sample selection bias [17]. These sampling methods were utilized to ensure the baseline cohort represented the sociodemographic background of 9–10-year-old children in the U.S.

Due to the COVID-19 pandemic, cardiometabolic risk measurement collection was disrupted due to the social distancing requirements of the COVID-19 pandemic, canceling non-essential research activities. Therefore, only 78.3 % of participants had their cardiometabolic risk measured at Year 2 follow-up. In Year 3 follow-up, only participants who were not able to have their cardiometabolic risks measured in Year 2 had their cardiometabolic risks measured. For this analysis, we combined cardiometabolic risk measures at Years 2 and 3 to include all participants in the ABCD study.

Hemoglobin A1c was collected for 1607 participants, TC was collected for 1476 participants, and HDL-C was collected for 1475 participants. The 10463 participants who were missing the three cardiometabolic risk measurements (hemoglobin A1c, TC, and HDL-C) or the six sociodemographic measurements were excluded from the data analyses (Appendix A). The current analysis included a sample of 1412 adolescents.

Study approval was given by the University of California, San Diego centralized Institutional Review Board, and the secondary data analysis approval was given by the University of California, San Francisco (UCSF). Written consent was given by caregivers/parents, and written assent was given by the study participants.

Age of the participant, household income (six categories), and parental education status (high school education or less vs. college education or more) were self-reported by the participants and/or their caregivers in Year 2. Sex assigned at birth (female or male), and race and ethnicity (Asian, Black, Latino/Hispanic, Native American, White, or other) were collected at baseline.

Blood samples were collected using standard venipuncture techniques and processed according to laboratory protocols [18]. Nonfasting measures included hemoglobin A1c, TC, and HDL-C. Non-HDL-C was calculated by subtracting HDL-C from TC.

Data analyses were performed using Stata 18 (StataCorp, College Station, TX). Descriptive statistics were calculated by measuring the mean, standard deviation, and percentages of the independent and dependent variables. Multivariable linear regression models were conducted to estimate the associations between the independent variables (age, sex, race and ethnicity, household income, and parental education) and continuous cardiometabolic risk factor outcomes (hemoglobin A1c, TC, HDL-C, and non-HDL-C).

## 3 Results

Of the total sample included (1412), 45.2 % of the participants were female and 38.5 % were non-White, with a mean age of 12.0 (±0.6) years. In our sample, 38.9 % had a household income of less than $75,000. The average hemoglobin A1c level was 5.2 % (±0.4 %), the average TC level was 156.6 (±28.9) mg/dL, the average HDL-C level was 56.0 (±12.9) mg/dL, and the average non-HDL-C level was 100.6 (±26.0) mg/dL (Table 1). Out of our sample, 0.5 % had diabetes (hemoglobin A1c ≥ 6.5 %), 7.6 % had high TC (≥200 mg/dL), and 7.4 % had low HDL-C (<40 mg/dL).

**Table 1:** Sociodemographic and metabolic characteristics of Adolescent Brain Cognitive Development (ABCD) Study participants (N = 1412).

Sociodemographic characteristics	Mean (SD)/n (%)
Age (years)	12.0 (0.6)
Sex, n (%)	
Female	638 (45.2 %)
Male	774 (54.8 %)
Race and ethnicity, n (%)	
Asian	59 (4.2 %)
Black	208 (14.7 %)
Latino/Hispanic	205 (14.5 %)
Native American	60 (4.2 %)
Other^a^	11 (0.8 %)
White	869 (61.5 %)
Household income, n (%)	
$24,999 or less	170 (12.0 %)
$25,000 to $49,999	177 (12.5 %)
$50,000 to $74,999	204 (14.4 %)
$75,000 to $99,999	204 (14.4 %)
$100,000 to $199,999	477 (33.8 %)
$200,000 or greater	180 (12.7 %)
Parent's highest education	
High school education or less	129 (9.1 %)
College education or more	1283 (90.9 %)
Cardiovascular disease risk measures	
Hemoglobin A1c (percent)	5.2 (0.4)
Total cholesterol (mg/dL)	156.6 (28.9)
HDL cholesterol (mg/dL)	56.0 (12.9)
Non-HDL cholesterol (mg/dL)	100.6 (26.0)
Diabetes status	
No diabetes (Hb A1C <6.5 %)	1405 (99.5 %)
Diabetes (Hb A1C ≥ 6.5 %)	7 (0.5 %)
Total cholesterol status	
Normal (<200 mg/dL)	1305 (92.4 %)
High total cholesterol (≥200 mg/dL)	107 (7.6 %)
HDL cholesterol status (mg/dL)	
Normal (≥40 mg/dL)	1307 (92.6 %)
Low HDL cholesterol (<40 mg/dL)	105 (7.4 %)

a This subcategory was listed as “other” but with no specific racial and ethnic groups defined, although write-ins were allowed.

The results of the linear regression models presented in Table 2 showed that male sex was associated with higher hemoglobin A1c (beta coefficient [B] 0.04; 95 % confidence interval [CI], 0.00, 0.08; p = 0.037) compared to female sex. Black race was associated with higher hemoglobin A1c (B 0.16; 95 % CI, 0.10, 0.22; p < 0.001) compared to White race, and Native American race was associated with higher hemoglobin A1c (B 0.14; 95 % CI, 0.05, 0.24; p = 0.003) compared to White race.

**Table 2:** Sociodemographic associations with metabolic the Adolescent Brain Cognitive Development (ABCD) Study (N = 1412).

Sociodemographic characteristics	Hemoglobin A1c		Total cholesterol		HDL cholesterol		Non-HDL cholesterol	
	B (95 % CI)	p	B (95 % CI)	p	B (95 % CI)	p	B (95 % CI)	p
Age (years)	−0.01 (−0.04, 0.02)	0.645	**−3.74 (-6.07, -1.41)**	**0.002**	**−1.06 (-2.08, -0.03)**	**0.043**	**−2.68 (-4.79, -0.58)**	**0.013**
Sex								
Female	reference		reference		reference		reference	
Male	**0.04 (0.00, 0.08)**	**0.037**	**−3.14 (-6.17, -0.11)**	**0.042**	−1.27 (−2.61, 0.06)	0.061	−1.86 (−4.60, 0.87)	0.182
Race and ethnicity								
Asian	0.05 (−0.04, 0.15)	0.250	1.55 (−6.06, 9.17)	0.689	**3.65 (0.30, 7.00)**	**0.033**	−2.10 (−8.98, 4.78)	0.550
Black	**0.16 (0.10, 0.22)**	**<0.001**	1.54 (−3.22, 6.29)	0.527	**3.07 (0.98, 5.17)**	**0.004**	−1.54 (−5.83, 2.76)	0.483
Latino/Hispanic	0.04 (−0.02, 0.09)	0.206	4.07 (−0.63, 8.78)	0.090	**2.70 (0.63, 4.77)**	**0.010**	1.37 (−2.88, 5.62)	0.527
Native American	**0.14 (0.05, 0.24)**	**0.003**	−2.95 (−10.66, 4.76)	0.454	0.08 (−3.31, 3.47)	0.962	−3.03 (−9.99, 3.93)	0.394
Other	−0.06 (−0.27, 0.15)	0.592	7.62 (−9.61, 24.86)	0.386	5.19 (−2.39, 12.76)	0.180	2.44 (−13.13, 18.00)	0.759
White	reference		reference		reference		reference	
Household income								
$24,999 or less	−0.01 (−0.10, 0.07)	0.738	**−10.31 (-17.08, -3.54)**	**0.003**	**−8.31 (-11.29, -5.33)**	**<0.001**	−2.00 (−8.11, −4.11)	0.521
$25,000 to $49,999	0.03 (−0.04, 0.11)	0.395	−6.12 (−12.58, 0.35)	0.064	**−7.11 (-9.95, -4.27)**	**<0.001**	0.99 (−4.85, 6.83)	0.739
$50,000 to $74,999	0.02 (−0.05, 0.10)	0.500	**−7.96 (-13.87, -2.06)**	**0.008**	**−3.22 (-5.81, -0.62)**	**0.015**	−4.75 (−10.08, 0.59)	0.081
$75,000 to $99,999	0.00 (−0.07, 0.07)	0.972	**−6.97 (-12.79, -1.16)**	**0.019**	−2.17 (−4.73, 0.39)	0.096	−4.81 (−10.06, 0.45)	0.073
$100,000 to $199,999	0.00 (−0.07, 0.6)	0.872	−3.08 (−8.03, 1.87)	0.222	**−2.28 (-4.46, -0.11)**	**0.040**	−0.80 (−5.27, 3.67)	0.725
$200,000 or greater	reference		reference		reference		reference	
Parent's highest education								
High school education or less	0.02 (−0.05, 0.09)	0.509	−0.25 (−6.07, 5.58)	0.933	−2.38 (−4.95, 0.18)	0.068	2.14 (−3.12, 7.40)	0.426
College education or more	reference		reference		reference		reference	

Bold indicates p < 0.05. B = coefficient from linear regression models. Models represent the abbreviated output from the linear regression models with covariate adjustment for age, sex, race and ethnicity, household income, and parent education.

Each one-year increase in age was associated with a 3.74 mg/dL (95 % CI, −6.07, −1.41; p = 0.002) decrease in TC. Male sex was associated with lower TC (B −3.14; 95 % CI, −6.17, −0.11; p = 0.042) compared to female sex. Household income of $24,999 or less, $50,000 to $74,999, and $75,000 to $99,999 were associated with lower TC compared to household income of $200,000 or greater.

Each one-year increase in age was associated with a 1.06 mg/dL (95 % CI, −2.08, −0.03; p = 0.043) decrease in HDL-C. Latino/Hispanic, Black, and Asian race and ethnicity were associated with higher HDL-C compared to White race. Household income of $24,999 or less, $25,000 to $49,999, $50,000 to $74,999, and $100,000 to $199,999 were associated with lower HDL-C compared to household income of $200,000 or greater.

Each one-year increase in age was associated with a 2.68 mg/dL (95 % CI, −4.79, −0.58; p = 0.013) decrease in non-HDL-C.

## 4 Discussion

In this analysis of a demographically diverse group of 10–14-year-old early adolescents in the U.S., several sociodemographic factors were associated with hemoglobin A1c and cholesterol, notably sex, race and ethnicity, and household income. Male sex was associated with higher hemoglobin A1c but lower TC. Black and Native American race and ethnicity were associated with higher hemoglobin A1c levels compared to White race. Asian, Black, and Latino/Hispanic were associated with higher HDL-C, in comparison to the White race. Higher household income was associated with higher TC and HDL-C. These findings from a diverse early adolescent population highlight sociodemographic disparities in cardiometabolic risk factors in early adolescents.

We found sex differences in hemoglobin A1c levels, with early adolescent boys having higher hemoglobin A1c levels compared to girls. These findings are consistent with prior research in U.S. children and young adults aged 5–24 years [19]. In contrast, our results demonstrated that early adolescent boys had lower TC compared to girls; there was also a trend of lower HDL-C in early adolescent boys compared to girls, but the difference was not statistically significant. This finding was consistent with the U.S. national data among 6–19 year-olds that girls had higher prevalence of high TC than boys [1]. Differences in cholesterol may be explained by the release of sex steroids, as the effects of estrogen and testosterone are thought to impact lipid profiles [20].

Black and Native American early adolescents had higher hemoglobin A1c levels when compared to White early adolescents. Similar results were observed in prior studies that showed non-Hispanic Black children and adolescents having higher hemoglobin A1c values than their White counterparts [21,22], but these studies included wider age ranges from 4 to 24 years. Moreover, Native American adults have been reported to show the highest prevalence of diabetes in the U.S. among all other racial and ethnic minority groups [23,24]. In general, racial minorities may have a greater risk of diabetes due to barriers to accessing healthcare, structural racism, stressors, disparities in public awareness and education, and geographical inequity [[25], [26], [27], [28]]. However, HDL-C was higher in Black, Latino/Hispanic, and Asian adolescents compared to their White counterparts. Our finding for Black adolescents is consistent with prior research finding higher HDL-C in Black compared to White adolescents and adults [28]. Explanations for these differences are unclear but could be due to differences in body composition (e.g., visceral adiposity) and physical activity [29,30].

Our finding that higher household income was generally associated with higher HDL-C compared to lower household income was consistent with prior findings in adults [31] and could be explained by lifestyle habits, such as diet quality and exercise frequency [30,32]. Our finding was also consistent with a study reporting that higher socioeconomic status (SES) was associated with higher HDL-C in urban female adults but inconsistent with a finding from the same study reporting that higher SES was associated with lower HDL-C in urban male adults [33]. Higher income levels generally allow for access to higher-quality foods and the opportunity for regular exercise patterns [31]. Adolescents with lower income backgrounds are more likely to consume fast food, yet less likely to engage in physical activity, overall contributing to lower HDL-C levels [31]. Our finding that higher household income was associated with higher TC could be driven by HDL-C. There was no association between household income and non-HDL-C.

Several interventions and primary prevention strategies have been previously identified to prevent the cardiometabolic risks associated with metabolic syndrome. Prior literature suggests that health messaging, which emphasizes overall wellness and not just avoiding risk, may be effective for adolescents [34]. The Life’s Essential 8 campaign by the American Heart Association recommends managing weight, blood sugar, blood pressure, and cholesterol alongside primary prevention measures such as promoting a healthy diet, regular physical activity, avoiding nicotine exposure, and maintaining good sleep health to improve and sustain cardiovascular health [35]. Primary care providers are well-positioned to deliver such interventions targeting risk factors including physical activity and diet [36]. School- and home-based lifestyle interventions with a focus on physical activity and nutrition have been shown to effectively reduce risk factors for metabolic syndrome for adolescents from ethnic minorities [37]. Intervention programs conducted within colleges, workplaces, churches, and community organizations should also be considered [[38], [39], [40], [41], [42], [43]]. Finally, digital interventions delivered via websites, social media, text messaging, email, and games are emerging strategies for reducing the cardiometabolic risks associated with metabolic syndrome [44,45].

The strengths of this study are the sociodemographically diverse sample of early adolescents. Limitations of this study should also be noted. Due to the cross-sectional nature, causality cannot be determined for our reported associations. One significant limitation pertains to the significant amount of missing data among participants due to various factors such as participant non-response, technical issues at the sites, and the COVID-19 pandemic, which could lead to selection and attrition bias. Given the smaller analytic sample size, there may be a greater margin of error with wider confidence intervals and results may have more limited generalizability. Future research could analyze cardiometabolic data in subsequent years after the COVID-19 pandemic when more data are available. Another limitation is that we could not control for study site after excluding participants with missing sociodemographic and metabolic data due to the small sample size.

## 5 Conclusions

Our findings explore sociodemographic differences in hemoglobin A1c and cholesterol levels among a diverse population of early adolescents, which can inform clinical and public health interventions to prevent these cardiometabolic risk factors. These findings may highlight key early adolescent populations to target for the Life’s Essential 8 campaign by the American Heart Association, which recommends managing weight, blood sugar, blood pressure, and cholesterol alongside primary prevention measures such as promoting a healthy diet, regular physical activity, avoiding nicotine exposure, and maintaining good sleep health to improve and sustain cardiovascular health [35]. Furthermore, early screening and regular monitoring of biomarkers such as hemoglobin A1c and cholesterol in healthcare settings, particularly for high-risk groups such as Black or Native American adolescents or those from low-income households, could improve the effectiveness of targeted interventions and prevent the progression to full metabolic syndrome or future cardiovascular outcomes [46].

## CRediT authorship contribution statement

**Jason M. Nagata:** Writing – review & editing, Writing – original draft, Formal analysis, Data curation, Conceptualization. **Christiane K. Helmer:** Writing – review & editing, Writing – original draft, Formal analysis. **Jennifer H. Wong:** Writing – review & editing, Writing – original draft, Formal analysis. **Seohyeong Lee:** Writing – review & editing, Writing – original draft. **Sydnie K. Domingue:** Writing – review & editing, Writing – original draft. **Patrick Low:** Writing – review & editing, Writing – original draft. **Abubakr A.A. Al-shoaibi:** Writing – original draft, Formal analysis, Data curation, Conceptualization. **Joan E. Shim:** Writing – review & editing, Writing – original draft, Formal analysis, Conceptualization. **Kyle T. Ganson:** Writing – review & editing. **Alexander Testa:** Writing – review & editing. **Orsolya Kiss:** Writing – review & editing, Conceptualization. **Holly C. Gooding:** Writing – review & editing, Conceptualization. **Erin E. Dooley:** Writing – review & editing, Conceptualization. **Kelley Pettee Gabriel:** Writing – review & editing, Conceptualization. **Fiona C. Baker:** Writing – review & editing, Data curation.

## Ethics approval

The University of California, San Diego provided centralized institutional review board (IRB) approval and each participating site received local IRB approval.

## Consent, data, materials and/or code availability

Written informed consent and assent were obtained from the parent/guardian and adolescent, respectively, to participate in the ABCD Study. Data used in the preparation of this article were obtained from the ABCD Study (https://abcdstudy.org), held in the NIMH Data Archive (NDA). Investigators can apply for data access through the NDA (https://nda.nih.gov/).

## Funding

J.M.N. was funded by the 10.13039/100000002National Institutes of Health (K08HL1549350 and R01MH135492) and the 10.13039/100000862Doris Duke Charitable Foundation (2022056). The funders had no role in the design and conduct of the study; collection, management, analysis, and interpretation of the data; preparation, review, or approval of the manuscript; and decision to submit the manuscript for publication.

The ABCD Study was supported by the 10.13039/100000002National Institutes of Health and additional federal partners under award numbers U01DA041022, U01DA041025, U01DA041028, U01DA041048, U01DA041089, U01DA041093, U01DA041106, U01DA041117, U01DA041120, U01DA041134, U01DA041148, U01DA041156, U01DA041174, U24DA041123, and U24DA041147. A full list of supporters is available at https://abcdstudy.org/federal-partners/. A listing of participating sites and a complete listing of the study investigators can be found at https://abcdstudy.org/principal-investigators.html. ABCD consortium investigators designed and implemented the study and/or provided data but did not necessarily participate in the analysis or writing of this report.

## Declarations of competing interest

None.
