# Supplementary material for: Social epidemiology of cardiometabolic risk factors in early adolescents
Source: Int J Cardiol Cardiovasc Risk Prev. 2025 Mar 6;25:200382. doi: 10.1016/j.ijcrp.2025.200382 (PMC11957581; doi:10.1016/j.ijcrp.2025.200382)
Supplement: Multimedia component 1 [file mmc1.docx]

| Appendix A. Comparison of the sociodemographic characteristics of the Adolescent Brain Cognitive Development (ABCD) study participants included vs. excluded in the analysis (N=11,875) | | | |
| --- | --- | --- | --- |
| Sociodemographic characteristics | Included (n=1,412) | Excluded (n=10,463) | p |
| Age (years) | 12.0 (0.6) | 12.0 (0.6) | 0.621 |
| Sex (%) |  |  | 0.396 |
| Female | 45.2% | 48.4% |  |
| Male | 54.8% | 51.6% |  |
| Race and ethnicity (%) |  |  | 0.002 |
| Asian | 4.2% | 2.1% |  |
| Black | 14.7% | 24.6% |  |
| Latino / Hispanic | 14.5% | 8.6% |  |
| Native American | 4.2% | 5.9% |  |
| Other | 0.8% | 1.6% |  |
| White | 61.5% | 57.2% |  |
| Household income (%) |  |  | 0.976 |
| $24,999 or less | 12.0% | 12.8% |  |
| $25,000 to $49,999 | 12.5% | 10.7% |  |
| $50,000 to $74,999 | 14.4% | 13.4% |  |
| $75,000 to $99,999 | 14.4% | 16.1% |  |
| $100,000 to $199,999 | 33.8% | 33.6% |  |
| $200,000 or greater | 12.7% | 13.4% |  |
| Parent's highest education (%) |  |  | 0.613 |
| High school education or less | 9.1% | 10.3% |  |
| College education or more | 90.9% | 89.7% |  |
